# Metabonomic Strategy to the Evaluation of Chinese Medicine Compound Danshen Dripping Pills Interfering Myocardial Ischemia in Rats

**DOI:** 10.1155/2013/718305

**Published:** 2013-05-02

**Authors:** Xue Xin, Haimiao Zou, Ningning Zheng, Xinchun Xu, Yinmin Liu, Xiaoxian Wang, Hongbing Wu, Lina Lu, Jing Su, Mingfeng Qiu, Xiaoyan Wang

**Affiliations:** ^1^School of Pharmacy, Shanghai Jiao Tong University, Shanghai 200240, China; ^2^Research Center for Traditional Chinese Medicine and Systems Biology, Shanghai University of Traditional Chinese Medicine, Shanghai 201203, China; ^3^Xuhui District Central Hospital of Traditional Chinese Medicine, Shanghai 200031, China; ^4^Ministry of Education, Key Laboratory of Systems Biomedicine, Shanghai Center for Systems Biomedicine, Shanghai Jiao Tong University, Shanghai 200240, China

## Abstract

Coronary heart disease (CHD) is one of the highest mortality diseases in the world. Traditional Chinese medicine compound Danshen dripping pills (CDDPs) have currently made a great achievement in treating CHD. However, the therapeutic mechanism of CDDP is often poorly interpreted. In this study, a GC-MS-based metabonomic study was conducted to assess the holistic efficacy of CDDP for myocardial infarction in male Sprague-Dawley rats, which were divided into the control group, the sham group, the model group, the control + CDDP group, and the model + CDDP, with CDDP at a dose of 107 mg/kg·d (equal to 1.8 mL/kg·d). The metabonomic findings demonstrated great differences of metabolic pattern among sham, model, and the model + CDDP in the orthogonal partial least squares discriminant analysis (OPLS-DA) models, which coordinated well with the assessment of plasma biochemistry and histopathological assay. Differentially expressed metabolites suggested that energy metabolism, glycolysis, and lipid metabolism might be disrupted by myocardial infarction. Both the potential metabolic biomarkers and the biochemical histopathological indices were regulated effectively by CDDP.

## 1. Introduction

Coronary heart disease (CHD) is caused by atherosclerotic coronary artery stenosis, leading to insufficient blood supply of coronary, myocardial ischemia (MI), and heart attack. CHD, including its most serious complication MI, is the most common cause of death in industrialized countries, and the morbidity is increasing dramatically in developing countries [1]. The effectiveness of current antiischemic medicines, for example,  *β*-blockers or calcium channel blockers, nitroglycerin, and angiotensin inhibitors, is limited by their side effects, such as hypotension and bradycardia [2]. With little side effect, significant effect, and a unique medical theory, especially by hitting multiple targets with the combination and compatibility of multicomponents drugs, traditional Chinese medicine (TCM) is getting more recognition in current researches.

Compound Danshen dripping pills (CDDPs,* Fufang Danshen Diwan* in Chinese) are developed on the basis of TCM theory and modern preparation technologies, consisting of *Radix salvia miltiorrhizae, Radix notoginseng*, and* Borneolum* [3]. With its multi-components, multieffects, and multitargets characters, CDDPs are generally applied in the prevention and treatment of coronary arteriosclerosis, angina pectoris, and hyperlipaemia in China [4], which leads to phase II clinical research in the USA in 2010 [5]. However, the widely employment of CDDPs is still facing a bottleneck in the United States of America and some European countries, not only because of the complex compounds, but also because of lack of an overall systemic evaluation and a standard to refer.

The emergency of metabolomics/metabolomics technology, which applies multivariate statistical techniques to analyze the highly complex data sets generated by advanced analytic instruments, such as nuclear magnetic resonance (NMR) and mass spectrometry (MS), has provided an opportunity for the resolution of this issue [6]. Metabonomics reflects the function of organisms from the end products of the metabolic network and the metabolic changes of a complete system caused by interventions in a holistic context [7]. This property accords with the holistic thinking of TCM, suggesting that metabonomics has a potential impact on our understanding of the theory behind the evidence-based TCM [8]. Consequently, metabonomics has been successfully utilized in understanding many aspects of TCM, such as the analysis of Chinese herbs [9], Chinese medicine syndromes [10], the mechanisms of Chinese medicine [11], and safety assessment [12].

Up to date, global metabolic profiling of biofluids (e.g., urine and sera) has been used meaningfully in understanding and biomarker detection of CHD [13], especially in the early diagnosis [14] and classification of Chinese syndromes of CHD [15, 16]. In the present study, combined with conventional pharmacological approaches, the metabonomic method is sought to be applied to investigate the change of endogenous metabolites in MI rats based on coronary artery ligation.

The finding of biopathways and potential biomarkers will be helpful in clarifying the mechanism of CDDP in treating MI.

## 2. Materials and Methods

### 2.1. Chemicals and Animals

HPLC grade methanol was purchased from Merck; HPLC grade chloroform and pyridine were purchased from Tedia. The following compounds were obtained from Sigma-Aldrich (St. Louis, MO, USA): O-methoxamine-HCL, bis (trimethylsilyl)-trifluoroacetamide (BSTFA, with 1% trimethylchlorosilane, TMCS), heptadecanoic acid, L-2-chlorophenylglycine, myristic acid, and urease. The assay kits for creatine kinases (CK), superoxide dismutase (SOD), and malondialdehyde (MDA) were purchased from Nanjing Jiancheng Bioengineering Institute (Nanjing, China). CDDP was purchased from Tasly Pharmaceutical Co., Ltd. (Tianjin, China). Ultrapure water (18.2 M*Ω*) was purified with a Milli-Q system (Millipore, MA, USA). All other chemicals used were of analytical grade.

 Male Sprague-Dawley rats, 6 to 7 weeks of age with body weights of 180 ± 8 g, were purchased from Sino British Sippr/BK Lab. Animal Co., Ltd. All animals were kept in an animal room with free access to food and water under standard conditions of temperature (23 ± 2°C), humidity (60 ± 5%), and light (12 h dark/light cycle). The experiment animals were housed under the above conditions for a 2-week acclimation period. All animal studies complied with current ethical considerations with the approval (SYXK-2007-0025) of Shanghai Jiao Tong University.

### 2.2. Myocardial Infarction Study and Drug Administration

The myocardial infarction (MI) of rats was induced by ligation of the left ventricular coronary artery [17]. 90 rats were randomly selected to undergo MI or sham surgery. First, animals were anesthetized with chloral hydrate (30 mg/kg, i.p.), fixed on the operating table while their backs were placed and intubated with a 16-gauge plastic catheter, which was connected with an animal breathing machine (rate: 85/min, respiratory ratio: 1 : 1, and volume: 18 mL), and electrocardiograms (ECG) was monitored by MPA 2000 biosignal analysis system (Alcott Biotech Co., Ltd., Shanghai, China) through electrocardioelectrode (lead II). Then, by separating subcutaneous tissue and muscle, thoracotomy was placed in the fourth left intercostal space. The heart was held gently outside the chest, while a silk suture was threaded beneath the left coronary artery 2-3 mm below the origin. After the heart had been returned to its normal position, the suture was ligated. The thorax was closed layer by layer under negative pressure, and a dose of penicillin (10,000 u/250 g, s.c.) was given one hour after surgery. Sham-operated rats were treated by using the similar manner except that coronary artery ligation was not done on them. 

 During the 24 h after operation, 44 of the 75 MI rats died because of acute pumps failure or lethal arrhythmias; 31 animals survived and were randomly divided into model group and mod. + CDDP group. 4 out of 15 sham surgery animals died throughout the experiment. 20 rats without undergoing experiment were randomly divided to control group and con. + CDDP (10 each group). The CDDP was dissolved in a certain amount of water to a certain concentration of per milliliter of drug solution. The con. + CDDP group and mol. + CDDP group were treated with drug solution at a dose of 107 mg/kg·d (equal to 1.8 mL/kg·d). Meanwhile, control group, sham group, and model group were treated with the same volume of 0.9% saline solution. The five animal groups were consecutively administrated as above for 28 days. During the administrated period, one rat in model group died.

### 2.3. Sample Collection and Preparation

On the day before operation (day-1) and days 3, 14, and 28, blood samples were collected from the vena ophthalmica, added, respectively, into 2.5 mL heparin-coated tubes, and then centrifuged at 3500 rpm for 10 min at 4°C. The supernatant obtained was frozen immediately, stored at −80°C, and thawed before analysis. On the 29th day after operation, all rats were anesthetized and subjected to autopsy. Their hearts were taken out immediately; 3 hearts of each group were fixed in 10% formalin, and the others were stored at −80°C.

### 2.4. Cardiac Enzymology and Histopathology

Plasma concentrations of CK were measured by ultraviolet spectrophotometer at 660 nm. The hearts were ground and diluted for 10 times in 0.9% saline solution at 0°C and then centrifuged at 3500 rpm for 10 min; the supernatant concentrations of superoxide dimutse (SOD) and malondialdehyde (MDA) were measured at 550 nm and 532 nm, respectively. Heart samples fixed in formalin were fixed and embedded in paraffin wax; 4-5 *μ*m of histologic sections of tissues was stained with hematoxylin-eosin. The sections were examined under microscope, and photomicrographs were taken. 

### 2.5. GC-MS Spectral Acquisition of Plasma Samples and Data Pretreatment

Plasma metabolites were subjected to trimethylsilyl derivatization and analyzed by gas chromatograph-mass spectrometer (GC-MS) [18]. Two internal standard solutions (10 *μ*L of L-2-chlorophenylalanine in water, 0.3 mg/mL; 10 *μ*L of heptadecanoic acid in methanol, 1 mg/mL) were spiked into a 100 *μ*L thawed aliquot of plasma sample. A mixture of methanol/chloroform (3 : 1) (300 *μ*L) was used to extract the metabolites from the plasma. After a vortexing period of 30 s and storage for 10 min at −20°C, the samples were centrifuged at 12000 rpm for 10 min. An aliquot of the 300 *μ*L supernatant was vacuum-dried at room temperature in a glass vial. The residue was derived with 80 *μ*L of methoxyamine (15 mg/mL in pyridine) at 30°C for 90 min and followed by 80 *μ*L of BSTFA (1%TMCS) at 70°C for 60 min. 

Each 1 *μ*L of solution of derivatives was injected into an Agilent 6890 GC-MS at 260°C in splitless mode. Metabolite separation was achieved on a DB-5 ms capillary column (30 m × 250 *μ*m i.d., 0.25 *μ*m film thickness; Agilent J&W Scientific, Folsom, CA, USA) with helium as the carrier gas at a constant flow rate of 1.0 mL/min. The GC oven temperature programming was started at 80°C and maintained for 2 min, followed by 10°C/min ramps to 180°C, 5°C/min to 240°C, and 25°C/min to 290°C and a final 9 min maintenance at 290°C. The temperature of transfer interface and ion source was set to 280 and 230°C, respectively. Electron impact ionization (70 eV) was used. Mass data was collected after a 5 min solvent delay at full scan mode (*m*/*z* 30–550) with an acquisition ate of 20 spectrum/second. 

All GC/MS files were converted to CDF format via Agilent MSD software. CDF files were processed with XCMS in R 2.7.2 (Free Software Foundation, Inc.) for data pretreatment procedures, such as baseline correction, smoothing and alignment, time-window splitting, and peak feature extraction. Three-dimensional output data results were obtained with arbitrary peak index information (retention time *m*/*z* pairs), sample names (observations), and peak intensity (variables). 

### 2.6. Data Processing

The resulting three-dimensional data sets from GC-MS analysis were introduced into SIMCA-P 11.5 software (Umetrics, Umeå, Sweden) for multivariate statistics [19]. Prior to multivariate statistics, the data was mean-centered and pareto-scaled. The unsupervised principal component analysis (PCA) was first used in all samples to see the general separation and find the outliers. Then, supervised partial least squares (PLS) and orthogonal partial least squares discriminant analyses (OPLS-DA) were performed. Plasma differential variables associated with MI and the effect of CDDP were selected based on a threshold of variable importance in the projection (VIP) value (VIP > 1) from a typical 7-fold cross-validated OPLS-DA model. In parallel, these differential metabolites from the OPLS-DA model were validated at a univariate level using Student's *t*-test. The critical *P* value of the test was set to 0.05 in this study. The corresponding fold change showed how the metabolites varied from MI compared with sham group. Compound identification was performed in NIST library 2005, with a similarity threshold of 70%. The analysis of the metabolites pathway was based on Kyoto Encyclopedia of Genes and Genomes (KEGG).

## 3. Result

### 3.1. Plasma Enzymes and Histological Assay

#### 3.1.1. Electrocardiograms and Cardiac Enzymology

**Figure 1:**
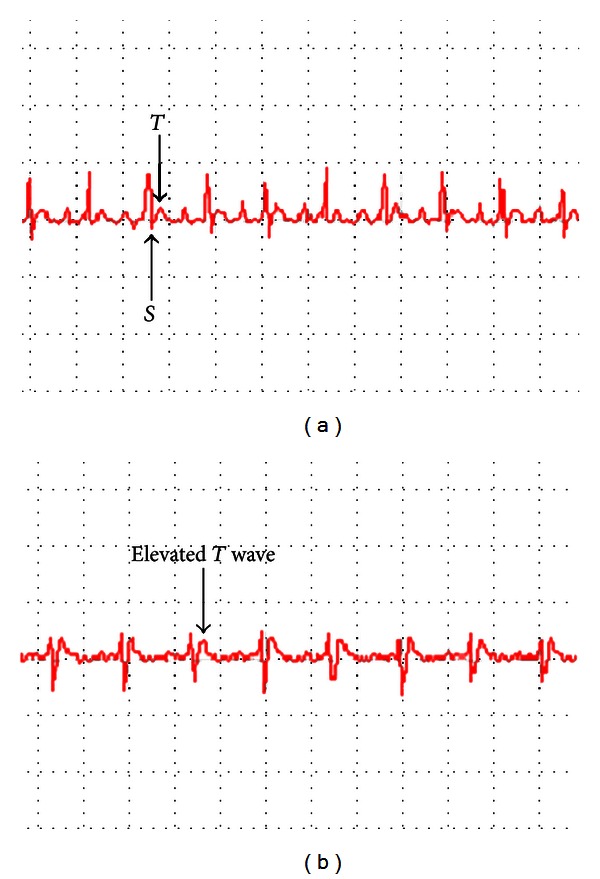
Electrocardiogram before (a) and after (b) coronary artery ligation.

The changes of T wave and S-T segment of ECG are generally considered as a main index to evaluate animal model of MI [20]. As shown in Figure 1, we found that T waves of ECG in MI model rats were inversed after ligation. Enzymes and biochemical materials in plasma or tissue, such as CK, SOD, and MDA, are cited as important parameters in the assessment of MI [21–24]. On day 3, the level of CK in plasma revealed a significant elevation in the model group compared to the controls and the sham surgery group (Table 1), suggesting the success of the MI model after coronary artery ligation. There was no statistical significance (*P* > 0.05) in the comparison between the control group and con. + CDDP, demonstrating that CDDP had little effect upon health rats. And a statistically significant restoration in CK level was observed in mod. + CDDP group compared with model group (*P* < 0.05), indicating that the effect of CDDP in recovering the plasma enzymes emerged within 3 days. On day 28, when compared with the control group, the concentrations of MDA (*P* < 0.05) in model group were significantly increased, while the levels of SOD (*P* < 0.05) were remarkably decreased. Restoration of the concentrations of SOD and MDA was observed in mod. + CDDP group, demonstrating the good performance of CDDP in treating MI after coronary artery ligation. 

#### 3.1.2. Effects of MI on Cardiac Tissue

**Figure 2:**
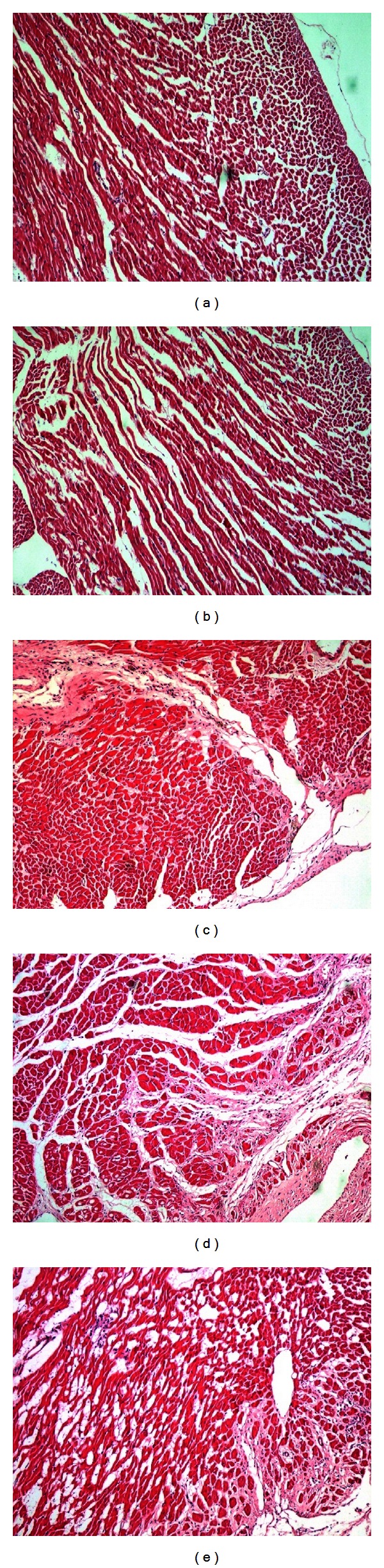
Myocardial tissue in light microscope (×100). (a) Control, (b) con. + CDDP, (c) sham, (d) model, and (e) mod. + CDDP.

After treatment, weight measurement of rats' hearts revealed statistical significance in the comparison among all groups (Table 2), demonstrating that the model group had little recovery after MI and CDDP had little effect upon health rats but exerted beneficial effects on MI rats greatly for rehabilitation. As shown in Figure 2, histopathological examination of myocardial tissue of control (Figure 2(a)) and con. + CDDP (Figure 2(b)) groups showed clear the orderly structure of heart without abnormality. In the sham group (Figure 2(c)), integrity of myocardial cell membrane was observed except a few myocardial cell necrosis and granulation tissue hyperplasia. However, in the model group (Figure 2(d)), there was serious necrobiosis with fibroblastic proliferation and presence of chronic inflammatory cells and lymphocytic infiltration. Moreover, edema and vacuolar, appeared along with absorption of myocardial cells near myocardial infarction areas, were clearly visible in the model group, whereas in the mod. + CDDP (Figure 2(e)), a reduction in inflammatory cells and less serious area of subendocardial damage was observed when compared with the model group. The above observations suggested that CDDP could decrease myocardial damage in MI rats but showed little influence on health rats.

### 3.2. Metabolomic Study of Plasma Samples

#### 3.2.1. PCA Processing of GC-MS Data

**Figure 3:**
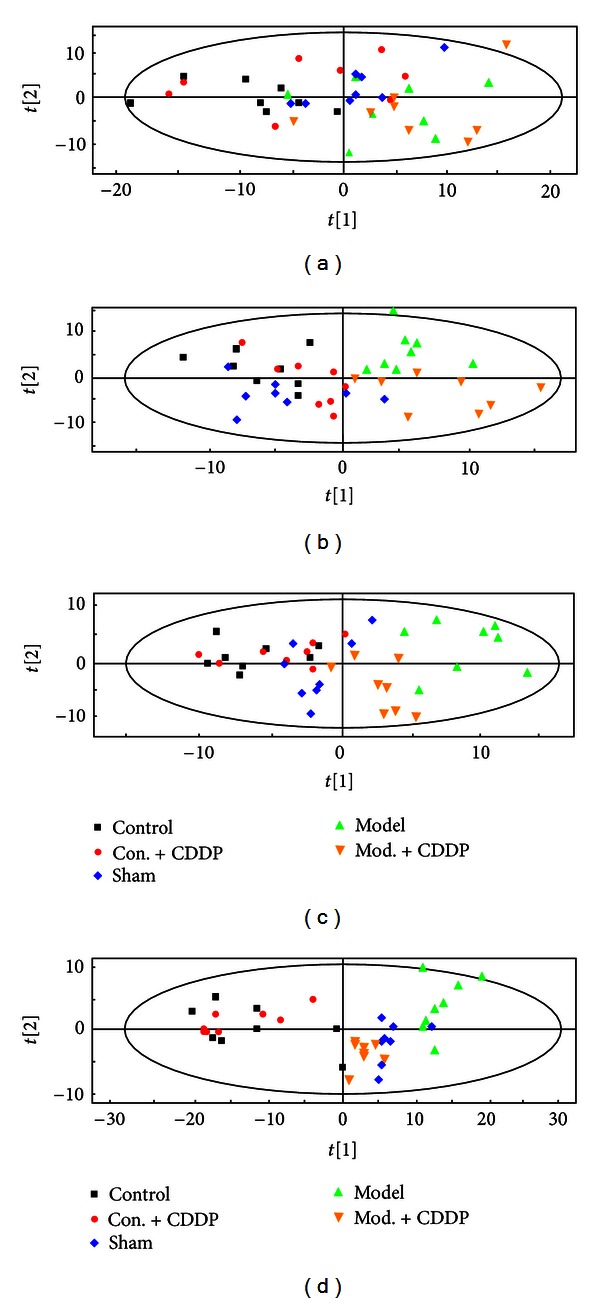
PCA scores plots of comparison of control, con. + CDDP, sham, model, and mod. + CDDP groups of rat plasma data on day-1 (a), day 3 (b), day 14 (c), and day 28.

Though the typical GC-MS total ion chromatograms (TIC) of plasma samples derived from each group, excluding internal standards, a total of 128 individual metabolites were consistently detected in nearly 90% of the plasma samples, including amino acids, organic acids, and amines. PCA approaches are frequently used to distinguish between classes expected to show metabolic differences. In this work, we performed PCA models of the plasma metabonomic data to exhibit classification of each group (as shown in Figure 3). In the PCA map, each spot represented a sample and each assembly of samples indicated a particular metabolic pattern of different groups. As shown in Figure 3(a), the metabolic state of each spot on day-1 mixed disorderly, which indicated that endogenous substances showed little difference in each group prior to MI surgery. On day 3 (Figure 3(b)), the metabolic state of model and mod. + CDDP groups was far away from the control, con. + CDDP, and sham position, and mod. + CDDP group could be classified with the model group, which suggested that MI altered the metabolic fingerprints of plasma compared to the normal state, and CDDP disturbed the endogenous substances compared with the model. On day 14 (Figure 3(c)) and day 28 (Figure 3(d)), the trajectory of mod. + CDDP group gradually gathered with the sham group, but the model group was still far away from them, revealing that CDDP had promoted the recovery of the disturbed metabolism state compared with the sham but still did not return to the normal state. The operation placed on rats also had influence on endogenous substances.

#### 3.2.2. Identification of MI-Related Metabolites

OPLS-DA, which is a common supervised pattern recognition to handle metabolomic data, picks out discriminating ions that are contributing to the classification of samples and remove noncorrelated variations contained within spectra. When the supervised pattern recognition was employed, the integrity of the mathematical model was evaluated first before being used for further interpretation. Commonly, R^2^Y provides an estimate of how well the model fits the Y data, whereas Q^2^Y is an estimate of how well the model predicts the Y. Both the Q^2^Y and R^2^Y close to 1 indicate an excellent model. 

**Figure 4:**
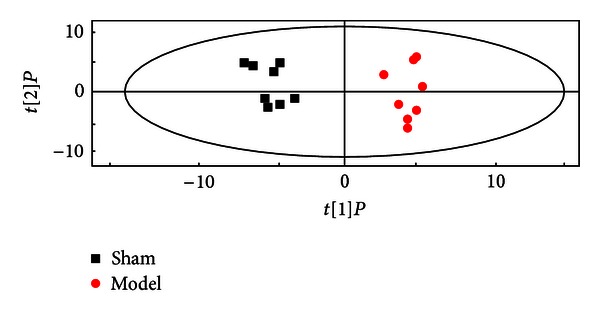
OPLS-DA score plot of GC-MS data from sham and model groups on day 3 (R^2^Y = 0.862, R^2^X = 0.264, and Q^2^Y = 0.569).

**Figure 5:**
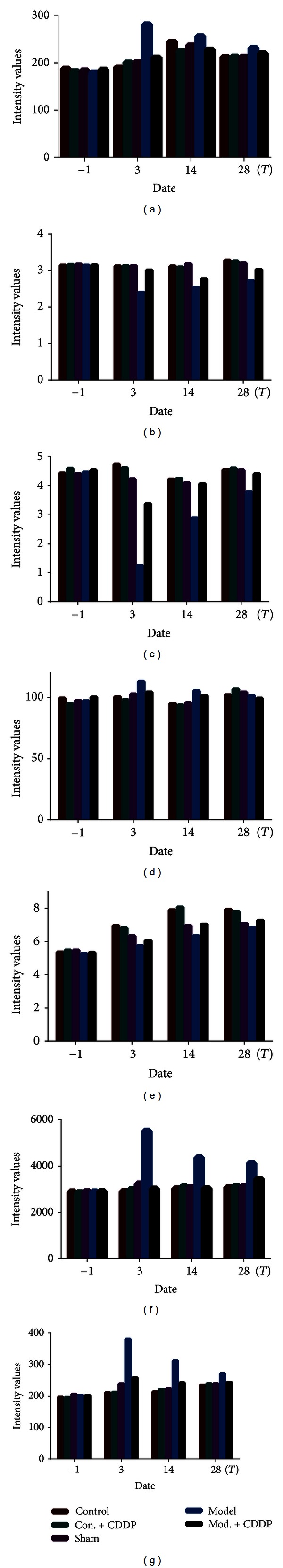
Summary of intensity values of urea (a), succinic acid (b), 2-butenedioic acid (c), L-aspartic acid (d), citric acid (e), D-glucose (f), and Lactic acid (g) in each group on each day.

 Thus, we used OPLS-DA models with satisfactory modeling and predictive abilities to compare between the sham and the model samples to find out biomarkers of MI in our study (Figure 4). Metabolites that significantly contributed to the clustering and discrimination were generated and identified according to 2.7. 20 significantly differential endogenous metabolites were selected for further study, and the results were listed in Table 3. The alterations of peak intensity of the 20 potential biomarkers of five groups were summarized in Table 4. Among the 20 metabolites, the levels of 11 compounds were observed significantly increased, and other 9 compounds were decreased in the model group. At the same time, 9 of the 20 metabolites, such as succinic acid, 2-butenedioic acid, creatinine, citric acid, D-glucose, urea, lactic acid, L-aspartic acid, and L-threonine, were observed significantly altered in the mod. + CDDP group compared to the model group. Therefore, these significantly differential endogenous metabolites in each group were assumed as potential biomarkers, which may be related to the diagnosis of MI and may be used as a potential efficacy indicator for drug effect.  Figure 5 was the alteration of peak intensity of each potential biomarker of five groups on each day. It was obvious that 7 listed biomarkers in mod. + CDDP group revealed recovery after the therapeutic intervention.

## 4. Discussion

The chemical analysis of CDDP reported previously shows that the main ingredients are salvianolic acids and sennosides [25, 26]. Salvianolic acids have been reported to improve cardiac function through improving antioxidant activity [27] and preventing cardiomyocyte apoptosis [28]. Senosides (including ginsenosides and notoginsenoside) could protect against myocardial damage and decrease lipid peroxidation [29]. By hitting multiple targets from a combination of multiple components, CDDP exerts the synergistic therapeutic efficacies of the prevention and treatment of cardiovascular diseases, including lowering blood fat, reducing blood viscosity, and resisting arteriosclerosis and antiplatelet aggregation [30].

The changes in the levels of CK, SOD, and MDA have been considered as important markers in the assessment of MI [21, 31]. Generation of oxygen-free radicals and subsequent formation of reactive oxygen species, which may cause lipid peroxidation and oxidative stress, has been implicated as a major causative factor for myocardial injury [22]. SOD is one of the antioxidants that can protect the heart from peroxidation, whoseconsequencedamages the tissues by inactivating the oxygen-free radicals. MDA is a poisonous end-product of lipid peroxidation, whose level can represent the rate and extent of lipid peroxidation directly and show the capability of eliminating free radicals indirectly [32]. In this study, the significant alternation in the levels of CK, SOD, and MDA was observed in the comparison between the model and the sham group, as well as the mod. + CDDP and the model group. These findings were in conformity with previous reports and demonstrated that myocardial ischemia was established in rats, and that CDDP had virtual significance in treating MI [1].

**Figure 6:**
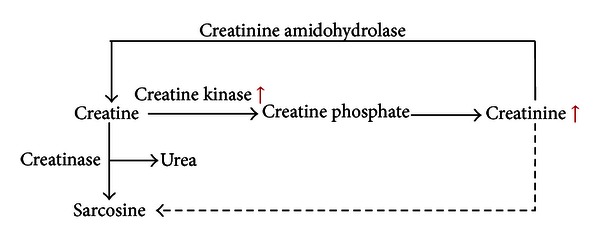
Creatinine and creatine in arginine and proline metabolism.

**Figure 7:**
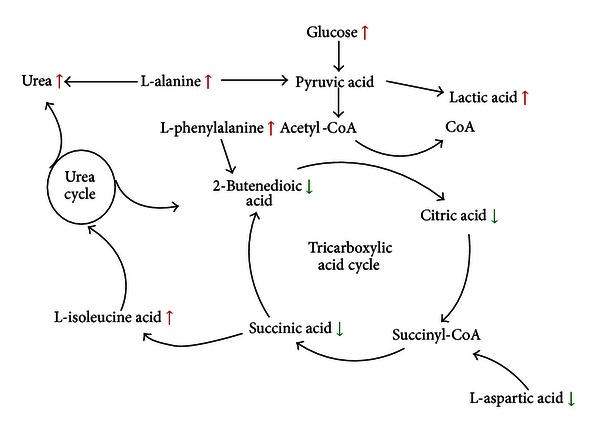
Metabolomic network in plasma of rats with myocardial ischemia.

Combined with conventional assessment of drug effects, our GC-MS-based metabonomic approach was employed to capture the differentially expressed endogenous metabolites associated with MI. 20 differential metabolites in the plasma samples were identified. Table 3 showed that most of the metabolites bounded up with MI were involved in metabolic processes related to myocardial energy metabolism, especially the TCA cycle (citric acid, succinic acid, and 2-butenedioic acid) and glycolysis (lactic acid and glucose). After MI surgery, myocardial blood flow of rats became inadequate in oxygen supply, and myocardial ischemia was developed. MI led to reducing formation of adenosine triphosphate (ATP) via aerobic mechanisms and accelerated anaerobic ATP production by glycolysis [11]. Meanwhile, creatine supplied the shortage of ATP and transformed into creatine by creatine kinase (as shown in Figure 6). Fatty acids are the main fuel for the healthy heart, with a lesser contribution coming from the oxidation of glucose and lactate. Myocardial ischemia dramatically alters fuel metabolism, causing an accelerated rate of glucose conversion to lactate and a switch from lactic acid uptake by the heart to lactate production. This causes a dramatic disruption in cell homeostasis (e.g., lactate accumulation and a decrease in pH and ATP) [33]; the lactate acid represents the end product of anaerobic or nonoxidative glycolysis and has been used as a marker of ischemia [34]. As shown in Figure 7, under the condition of MI, the levels of citric acid, succinic acid, and 2-butenedioic acid in tricarboxylic acid (TCA) cycle were decreased, whereas the lactic acid via glycolysis was increased. Meanwhile, the TCA cycle was not only the pathway of sugar decomposition but also the pathway of fuel molecules oxidation, such as fatty acids and amino acids. Therefore, the reduction of TCA cycle intermediates led to the alteration of fatty acid and amino acid metabolites, including L-alanine, phenylalanine, L-aspartic acid, L-isoleucine, and creatinine. After the administration of CDDP, the levels of these MI-related metabolites, citric acid, lactic acid, succinic acid, 2-butenedioic acid, and L-aspartic acid, in the mod. + CDDP group, were restored to the normal status (not significant with the sham group), suggesting that CDDP had exerted its therapeutic efficacies by regulating metabolites disrupted by MI.

## 5. Conclusion

In this work, GC-MS-based metabonomic strategy was used to investigate therapeutic effects of CDDP in treating MI. Combined with serum biochemistry and histopathological assessment such as assay, our metabonomic findings suggested that CDDP exerted its therapeutic efficacies by regulating energy metabolism, glycolysis, and lipid metabolism disrupted by MI. 

## Figures and Tables

**Table 1:** CK, SOD, and MDA content of rats (mean ± sd, *n* = 6).

Group	CK (U/mL)	SOD (U/mL)	MDA (nmol/mg prot)
Control	0.756 ± 0.18^a^	330.5 ± 78.8^a^	0.728 ± 0.122^a^
Con. + CDDP	0.837 ± 0.13^a^	330.4 ± 20.6^a^	0.832 ± 0.131^a^
Sham	0.748 ± 0.24^a^	323.5 ± 46.3^a^	0.833 ± 0.174^a^
Model	1.144 ± 0.44^b^	279.4 ± 102.9^b^	1.430 ± 0.468^b^
Mod. + CDDP	0.851 ± 0.35^a^	334.0 ± 29.0^a^	0.884 ± 0.094^a^

CK: creatine kinases; SOD: superoxide dismutase; MDA: malondialdehyde. compared with the model group, ^a^ *P* < 0.05; Compared with the control group, ^b^ *P* < 0.05.

**Table 2:** The weight of rats, heart, and heart-body ratio (mean ± sd, *n* = 6).

Group	Body weight (g)	Heart weight (g) (nmol/mg prot)	Heart-body ratio (mg/g) (U/mg prot)
Control	336.300 ± 33.853	1.015 ± 0.092^a^	3.029 ± 0.243^a^
Con. + CDDP	333.900 ± 40.932	1.005 ± 0.119^a^	3.020 ± 0.207^a^
Sham	349.636 ± 36.825	1.199 ± 0.179^b,a^	3.438 ± 0.420^b,a^
Model	339.000 ± 37.165	1.298 ± 0.102^b,c^	3.866 ± 0.461^b,c^
Mod. + CDDP	349.133 ± 41.461	1.204 ± 0.146^b,a^	3.470 ± 0.410^b,a^

Compared with the model group, ^a^ *P* < 0.05; compared with the control group, ^b^ *P* < 0.01; compared with the sham group, ^c^ *P* < 0.05.

**Table 3:** List of identified differential metabolites between the sham and model group on day 3.

tR (min)	Metabolites	VIP^a^	*P* ^b^	Fold change^c^ (model/sham)	Metabolic pathways
6.34	Glycine	1.6	0.0029	−1.1	Amino acid metabolism
7.14	Urea	1.4	0.0221	1.4	Nucleic acid metabolism
7.64	L-alanine	1.4	0.0134	1.3	Amino acid metabolism
8.72	L-isoleucine	2.5	0.0000	−1.1	Amino acid metabolism
8.9	Cadaverine	1.6	0.0058	2.1	Not known
9.01	Succinic acid	1.2	0.0532	−1.3	Energy metabolism
9.52	2-Butenedioic acid	1.9	0.0003	−3.4	Energy metabolism
9.96	L-threonine	2.0	0.0002	−1.1	Amino acid metabolism
11.06	Aminomalonic acid	1.3	0.0236	−1.2	Amino acid metabolism
11.69	L-aspartic acid	2.3	0.0000	1.1	Amino acid metabolism
11.72	L-proline	2.0	0.0002	−2.3	Amino acid metabolism
12.14	Creatinine	1.7	0.0010	1.2	Amino acid metabolism
13.04	L-phenylalanine	1.3	0.0192	1.4	Energy metabolism
16.18	Citric acid	1.3	0.0407	−1.1	Energy metabolism
16.86	D-xylose	1.2	0.0328	1.2	Nucleic acid metabolism
16.89	D-glucose	1.7	0.0017	1.7	Energy metabolism
17.19	Lactic acid	1.8	0.0010	1.6	Energy metabolism
17.58	L-tyrosine	1.7	0.0016	1.2	Amino acid metabolism
19.84	Propanoic acid	1.3	0.0138	−1.1	Energy metabolism
21.95	L-tryptophan	1.7	0.0012	1.1	Amino acid metabolism

^a^Variable importance in the projection (VIP) was obtained from OPLS-DA with a threshold of 1.0. ^b^ *P* values were calculated from Student's *t*-test. ^c^Fold change was calculated from the arithmetic mean values of each group. Fold change with a positive value indicates a relatively higher concentration present in model group while a negative value means a relatively lower concentration as compared to the sham group.

**Table 4:** Summary of intensity values of potential biomarkers in each group on day 3.

Biomarkers	Peak intensity (mean ± sd, *n* = 6)				
	Control	Con. + CDDP	Sham	Model	Mod. + CDDP
Glycine	5.86 ± 0.6^b,c^	5.51 ± 0.96^b^	4.85 ± 0.94^a^	4.41 ± 1.65^a^	5.94 ± 1.8^b,c^
Urea	190.9 ± 112.08^b^	201.44 ± 108.76^b^	201.59 ± 55.12^b^	282.23 ± 72.75^a,c^	211.97 ± 48.32^b^
L-alanine	444.97 ± 64.71^b^	432.48 ± 74.21^b^	450.96 ± 56.85^b^	586.25 ± 64.89^a,c^	443.53 ± 98.11^b^
L-isoleucine	260.88 ± 34.02^b,c^	254.42 ± 39.69^b^	211.84 ± 34.61^a,b^	192.58 ± 33.25^ac^	205.51 ± 54.06^a^
Cadaverine	22.75 ± 11.3^b^	23.12 ± 4.15^b^	21.68 ± 11.31^b^	45.53 ± 11.1^a,c^	36.9 ± 14.92^b^
Succinic acid	3.12 ± 2.16^b^	3.13 ± 0.94^b^	3.13 ± 1.63^b^	2.40 ± 1.01^a^	3.01 ± 2.07^b^
2-Butenedioic acid	4.74 ± 1.61^b^	4.61 ± 3.24^b^	4.23 ± 3.98^b^	1.24 ± 3.92^a,c^	3.37 ± 3.98^a,b^
L-threonine	209.33 ± 25.38^b,c^	208.3 ± 27.54^b^	181.25 ± 22.81^a,b^	164.77 ± 35.78^a,c^	189.33 ± 44.05^b^
Aminomalonic acid	46.73 ± 29.21^b^	46.21 ± 18.02^b^	43.69 ± 44.56^b^	36.41 ± 28.42^a,c^	39.85 ± 25.14^a^
L-aspartic acid	100.32 ± 11.51^b^	97.98 ± 13.89^b^	102.57 ± 7^b^	112.83 ± 13.89^a,c^	104.12 ± 21.12^b^
L-proline	280.57 ± 22.74^b,c^	289.33 ± 56.22^b^	218.72 ± 31.35^a,b^	95.1 ± 15.54^a,c^	248.84 ± 64.37^b^
Creatinine	6.39 ± 1.59^b,c^	6.27 ± 3.44^b^	7.25 ± 3.23^a,b^	8.7 ± 3.73^ac^	7.71 ± 3.24^a,b^
L-phenylalanine	110.09 ± 10.46^b,c^	106.57 ± 14.11^b^	126.76 ± 8.24^a,b^	177.46 ± 12.68^a,c^	148.95 ± 24.79^a,b^
Citric acid	6.95 ± 5.52^b^	6.83 ± 4.4^b^	6.33 ± 4.45^b^	5.75 ± 2.64^a,c^	6.06 ± 3.71^a,b^
D-xylose	7.01 ± 1.96^b,c^	7.08 ± 1.06^b^	8.13 ± 1.07^a,b^	9.76 ± 1.77^a,c^	8.07 ± 1.15^b^
D-glucose	2912.13 ± 190.45^b^	2993.74 ± 210.95^b^	3239.06 ± 255.96^b^	5506.4 ± 255.01^a,c^	3012.34 ± 209.33^b^
Lactic acid	210.17 ± 15.22^b^	211.91 ± 22.87^b^	238.35 ± 16.13^b^	381.36 ± 24.33^a,c^	258.68 ± 16.45^b^
L-tyrosine	44.57 ± 5.36^b,c^	43.47 ± 7.74^b^	56.62 ± 5.38^a,b^	67.94 ± 9.93^a,c^	62.72 ± 9.05^a^
Propanoic acid	21.98 ± 6.65^b,c^	22.5 ± 10.64^b^	11.98 ± 6.28^a^	10.89 ± 9.27^a^	14.96 ± 10.47^a,b^
L-tryptophan	421.7 ± 67.69^b^	417.94 ± 69.49^b^	434.8 ± 82.32^b^	478.28 ± 118.65^a,c^	451.78 ± 101.75^a,b^

Compared with the control group, ^a^ *P* < 0.05; compared with the model group, ^b^ *P* < 0.05; compared with the sham group, ^c^ *P* < 0.05.

## Abbreviations

CHD: Coronary atherosclerotic heart disease
MI: Myocardial ischemia
TCM: Traditional Chinese medicine
CDDP: Compound Danshen dripping pills
GC/MS: Gas chromatography/mass spectrometry
CK: Creatine kinases
SOD: Superoxide dismutase
MDA: Malondialdehyde
VIP: Variable importance in the project
PCA: Principal component analysis
PLS-DA: Partial least square-discriminant analysis
OPLS: Optimized potentials for liquid simulations
KEGG: Kyoto Encyclopedia of Genes and genomes
TIC: Total ion chromatograms
TCA: Tricarboxylic acid cycle.
